# Comparative cost-effectiveness of first-line pembrolizumab plus chemotherapy vs. chemotherapy alone in persistent, recurrent, or metastatic cervical cancer

**DOI:** 10.3389/fimmu.2023.1345942

**Published:** 2024-01-11

**Authors:** Ying-tao Lin, Chang Wang, Xiao-yan He, Qi-min Yao, Jian Chen

**Affiliations:** ^1^ Clinical Medical Research Center, Clinical Oncology School of Fujian Medical University, Fujian Cancer Hospital, Fuzhou, Fujian, China; ^2^ Department of Drug Clinical Trial Institution, Clinical Oncology School of Fujian Medical University, Fujian Cancer Hospital, Fuzhou, Fujian, China; ^3^ Department of Lymphoma & Head and Neck Tumors, Clinical Oncology School of Fujian Medical University, Fujian Cancer Hospital, Fuzhou, Fujian, China; ^4^ Department of Endocrinology, Fuqing City Hospital of Fujian, Fuqing City Hospital Affiliated to Fujian Medical University, Fuqing, Fujian, China; ^5^ College of Finance, Fujian Jiangxia University, Fuzhou, Fujian, China; ^6^ Department of Gynecological-Surgical Oncology, Clinical Oncology School of Fujian Medical University, Fujian Cancer Hospital, Fuzhou, Fujian, China

**Keywords:** cervical cancer, cost-effectiveness, partitioned survival model, pembrolizumab, chemotherapy

## Abstract

**Background:**

Treating persistent, recurrent, or metastatic cervical cancer remains challenging. Although pembrolizumab, combined with chemotherapy and bevacizumab, offers a promising first-line option, its cost-effectiveness within the Chinese healthcare system has not been established.

**Methods:**

A partitioned survival model was constructed using patient data from the KEYNOTE-826 trial. Efficacy, safety, and economic data from both trial and real-world practices were utilized to determine the costs, quality-adjusted life years (QALYs), and incremental cost-effectiveness ratio (ICER) of the treatment strategies. Comprehensive insights were gained through the sensitivity and subgroup analyses.

**Results:**

Over five years, the combination of pembrolizumab, chemotherapy, and bevacizumab offered an additional 1.18 QALYs compared to that provided by standard treatments. This regimen increased the costs by US$ 134,502.57, resulting in an ICER of US$ 114,275.67 per QALY, relative to traditional treatment costs. The ICER for the pembrolizumab regimen was further calibrated to be US$ 52,765.69 per QALY. Both ICER values surpassed China’s established willingness-to-pay threshold. Importantly, subgroup analysis revealed enhanced cost-effectiveness in patients presenting with a programmed death-ligand 1 combined positive score (PD-L1 CPS) ≥10.

**Conclusion:**

Introducing pembrolizumab alongside chemotherapy and bevacizumab may not be a cost-effective primary strategy for advanced cervical cancer against current standards. However, for patients with a PD-L1 CPS ≥10, the therapeutic and economic outcomes could be improved by adjusting the pembrolizumab price.

## 1 Introduction

Cervical cancer is the fourth leading cause of tumor-related mortality among women globally (1, 2). Remarkably, 80% of these cases are concentrated in developing nations, with China having the highest number of patients with cervical cancer worldwide (3). In China, the incidence of cervical cancer is 15.6%, with an annual increase of 4.1%. Notably, mortality rates are elevated in rural locales compared to those in urban areas, and the central and western provinces register rates that are approximately double those of the eastern provinces (4, 5). The members of two age-based cohorts of 40–50 and 60–70 years exhibited high susceptibility to cervical cancer in China, with the median age of diagnosis being 51 (6, 7). Cervical cancer screening, by cervical smear (Pap test) and Human Papilloma Virus test, is not widely carried out in the rural areas of China, and a large number of patients are diagnosed at an advanced stage (7, 8). Unfortunately, 80% of patients do not survive past five years of diagnosis (9, 10). Radical surgical intervention remains the primary treatment for the management of cervical cancer in early-stage patients, though the choice of surgical approach continues to be controversial (11, 12). Currently, an open approach is increasingly being proven as the preferred method for treating cervical cancer (13, 14). For advanced stages of the disease, cisplatin-based chemotherapy consistently stands as the cornerstone of systemic treatment, demonstrating significant efficacy. The landmark success of the GOG-240 trial in 2014 ushered in bevacizumab as a frontline therapeutic agent for cervical cancer, augmenting the overall survival (OS) by 3.5 months (15). The 2021 V1 iteration of the National Comprehensive Cancer Network (NCCN) guidelines endorses a combination of cisplatin-based chemotherapy and bevacizumab as the gold standard first-line therapeutic strategy for advanced cervical cancer. Non-surgical interventions, including radiotherapy, chemotherapy, targeted therapies, and notably, immunotherapy, are currently gaining research interest, with particular attention on immune checkpoint inhibitors (16). Based on the promising results of the KEYNOTE-826 trial, the United States Food and Drug Administration sanctioned the amalgamation of pembrolizumab and chemotherapy, with or without bevacizumab, for the primary treatment of recurrent or metastatic cervical cancer in patients with programmed death-ligand 1 (PD-L1)-positivity (with a combined positive score (CPS) ≥1). This combination regimen mitigated the mortality risk in patients with PD-L1-positive cervical cancer by 36% during the first-line treatment, significantly extending the OS and progression-free survival (PFS) (17). Nevertheless, the commendable therapeutic efficacy of pembrolizumab is shadowed by its exorbitant cost, imposing substantial economic strains on the healthcare infrastructure, which is more evident in rural areas where cervical cancer is prevalent and in the central-western parts of China. Pembrolizumab-related clinical trials have been emphasized for its clinical efficacy, leaving its cost-effectiveness largely uncharted. Hence, the present study aimed to dissect the economic implications of deploying a treatment regimen involving pembrolizumab for advanced cervical cancer from the vantage of the Chinese healthcare framework (18). This analysis seeks to evaluate both the clinical and economic efficacies of this intervention in the Chinese patient population, thereby providing crucial insights for patients, healthcare practitioners, and policymakers. Our study was designed by referring to the International Council for Harmonization E6 guidelines for Good Clinical Practice, Declaration of Helsinki principles, and applicable laws and regulations (19). The reporting criteria of the Consolidated Health Economic Evaluation Reporting Standards were followed when writing the economic evaluation section (20).

## 2 Materials and methods

### 2.1 Target population

The target population of our study was designed to mirror that of the KEYNOTE-826 trial. Participants were required to be adults, aged 18 or older, with an Eastern Cooperative Oncology Group (ECOG) performance-status score of either 0 or 1, reflecting their functional status. The study targeted a specific patient group: those with persistent, recurrent, or metastatic adenocarcinoma; adenosquamous carcinoma; or squamous cell carcinoma of the cervix. These patients had not previously undergone systemic chemotherapy and were not eligible for radical treatment. Furthermore, candidates must not have received any form of antineoplastic therapy, including chemotherapy or radiotherapy, within two weeks prior to this study. Any toxic reactions from previous treatments must have been fully resolved for inclusion of patients in the study.

In addition to these criteria, participants were required to present with diseases that could be assessed according to the Response Evaluation Criteria in Solid Tumors (RECIST), version 1.1. This ensured a standardized and objective assessment of the tumor response. Finally, the determination of PD-L1 status necessitated a biopsy. Although a newly procured biopsy was preferred, an archival tumor tissue sample, particularly from a non-irradiated lesion, was also acceptable.

### 2.2 Intervention

Patients were allocated in a 1:1 ratio to receive pembrolizumab (200 mg) combined with chemotherapy or chemotherapy only, with treatment administered every three weeks. The standardized chemotherapy regimen included paclitaxel (175 mg/m^2^ body surface area) and cisplatin (50 mg/m^2^). Additionally, based on the investigator’s judgment, bevacizumab was administered at a dose of 15 mg/kg body weight every three weeks. All the trial agents were delivered intravenously. Stratification during randomization considered the presence of metastatic disease at diagnosis (yes vs. no), intended use of bevacizumab (yes vs. no), and PD-L1 CPS category (<1, 1 to <10, and ≥10). Treatment persisted until the patient reached the maximum cycle count for each component, exhibited radiographic progression, experienced intolerable toxic effects, received prohibited therapies (e.g., novel antineoplastic treatments or non-palliative radiotherapy), or withdrew their consent or the investigator opted to terminate the regimen.

### 2.3 Model construction

**Figure 1:**
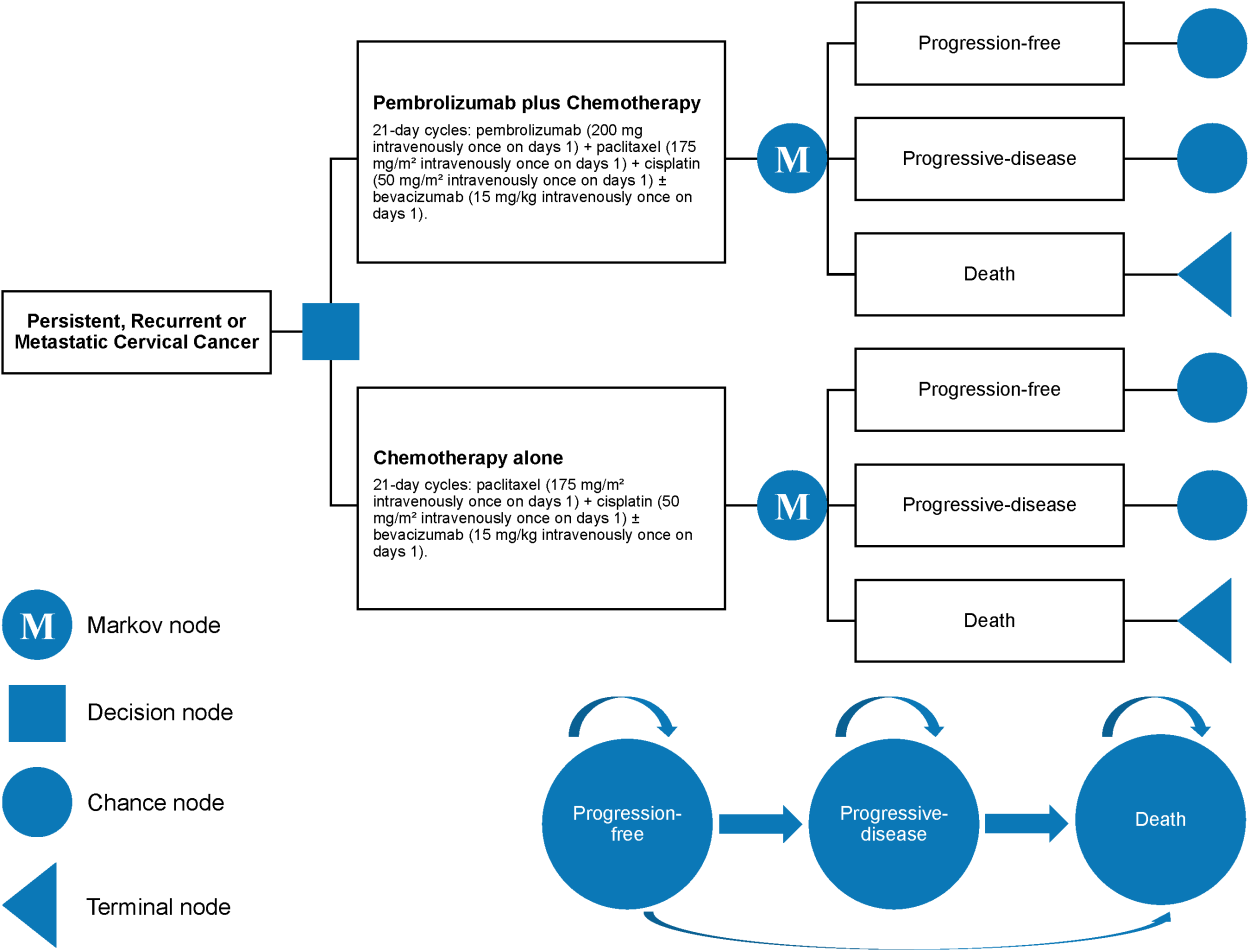
Partitioned survival model overview from the KEYNOTE-826 trial analysis.

The cost-effectiveness of the treatments was assessed using a partitioned survival model based on the data from the KEYNOTE-826 trial, a standard approach in metastatic oncology research (**Figure 1**). This model categorized the patient journey into three distinct health states: progression-free (from patient entry to disease progression), progressive disease (PD) (the duration from disease progression onset while the patient remains alive), and terminal (the point of patient death). Each cycle in the model lasted 3 weeks, with a 5-year time horizon (equivalent to 86 cycles), aimed at simulating the 5-year survival rate of patients with recurrent or metastatic cervical cancer who received pembrolizumab with chemotherapy or chemotherapy only. Key model outputs included cost, quality-adjusted life-years (QALYs), and incremental cost-effectiveness ratio (ICER).

### 2.4 Cost assessment

We integrated different clinical expenses associated with cancer treatment for the evaluation. These costs included drug acquisition, laboratory tests, radiological evaluations, medication administration, consultations related to disease progression, treatment-related adverse events (AEs), and end-of-life care expenses (terminal cost). We designated these as direct medical expenditures, converting them to US dollars using the August 2023 exchange rate of 1 USD = 7.2897 RMB. Our financial data were sourced from esteemed entities such as the National Health Commission of China, the Health Commission of Fujian Province, and expert consensus data. The detailed cost parameters are presented in **Table 1**.

**Table 1:** Key input parameters and their ranges used for sensitivity analysis in our model.

Input parameter	Base case value	Lower bound	Upper bound	Distribution	Source
Log-Logistic OS survival model					
Pembrolizumab plus chemotherapy	Scale (λ) = 24.217; Shape (γ) = 1.546	NA	NA	Log-logistic	(17)
Chemotherapy alone	Scale (λ) = 17.255; Shape (γ) = 1.718	NA	NA	Log-logistic	(17)
Log-logistic PFS survival model					
Pembrolizumab plus chemotherapy	Scale (λ) = 12.2045; Shape (γ) = 1.4758	NA	NA	Log-logistic	(17)
Chemotherapy only	Scale (λ) = 8.1898; Shape (γ) = 1.7748	NA	NA	Log-logistic	(17)
Drug acquisition, US$					
Pembrolizumab (Merck Sharp & Dohme Corp.) per 100 mg	2,457.99	1,966.39	2,949.59	Gamma	National Health Commission of China
Paclitaxel (Haikou Pharmaceutical Factory Co., Ltd.) per 30 mg	22.15	17.72	26.59	Gamma	National Health Commission of China
Cisplatin (Jiangsu Hossen Pharmaceutical Group Co., Ltd.) per 30 mg	2.62	2.10	3.15	Gamma	National Health Commission of China
Bevacizumab (Shanghai Henlius Biotech Inc.) per 100 mg	158.85	127.08	190.63	Gamma	National Health Commission of China
Drug administration, per cycle, US$					
Drug administration hospitalization	41.15	32.92	49.38	Gamma	Local medical data
Drug administration preventive medication	1.65	1.32	1.98	Gamma	Local medical data
Drug administration infusion	1.63	1.30	1.95	Gamma	Local medical data
Laboratory and imaging examination, US$					
12-lead ECG	3.70	2.96	4.44	Gamma	Fujian Provincial Health Commission
Hematology	3.43	2.74	4.12	Gamma	Fujian Provincial Health Commission
Serum chemistry	24.69	19.75	29.63	Gamma	Fujian Provincial Health Commission
Urinalysis	4.12	3.29	4.94	Gamma	Fujian Provincial Health Commission
Coagulation	9.13	7.30	10.96	Gamma	Fujian Provincial Health Commission
Thyroid	20.58	16.46	24.69	Gamma	Fujian Provincial Health Commission
Contrast-enhanced CT	336.09	268.87	403.31	Gamma	Fujian Provincial Health Commission
Costs of AE (Grade ⩾3), per cycle, US$					
Anemia	138.57	110.86	166.29	Gamma	expert consensus
Nausea	20.13	16.10	24.15	Gamma	expert consensus
Diarrhea	3.51	2.81	4.21	Gamma	expert consensus
Constipation	4.04	3.23	4.85	Gamma	expert consensus
Arthralgia	17.69	14.15	21.22	Gamma	expert consensus
Peripheral neuropathy	1.45	1.16	1.74	Gamma	expert consensus
Vomiting	20.13	16.10	24.15	Gamma	expert consensus
Hypertension	0.07	0.06	0.08	Gamma	expert consensus
Urinary tract infection	126.03	100.83	151.24	Gamma	expert consensus
Neutropenia	399.63	319.71	479.56	Gamma	expert consensus
Peripheral sensory neuropathy	1.45	1.16	1.74	Gamma	expert consensus
Thrombocytopenia	1,094.70	875.76	1,313.63	Gamma	expert consensus
Pembrolizumab plus chemotherapy AE risks (grade ⩾3)					
Anemia	0.303	0.242	0.36	Beta	(17)
Nausea	0.020	0.016	0.02	Beta	(17)
Diarrhea	0.020	0.016	0.02	Beta	(17)
Constipation	0.003	0.003	0.00	Beta	(17)
Arthralgia	0.007	0.005	0.01	Beta	(17)
Peripheral neuropathy	0.026	0.021	0.03	Beta	(17)
Vomiting	0.026	0.021	0.03	Beta	(17)
Hypertension	0.094	0.076	0.11	Beta	(17)
Urinary tract infection	0.088	0.070	0.11	Beta	(17)
Neutropenia	0.124	0.099	0.15	Beta	(17)
Peripheral sensory neuropathy	0.010	0.008	0.01	Beta	(17)
Thrombocytopenia	0.075	0.060	0.09	Beta	(17)
Chemotherapy alone AE risks (grade ⩾3)					
Anemia	0.269	0.215	0.32	Beta	(17)
Nausea	0.016	0.013	0.02	Beta	(17)
Diarrhea	0.026	0.021	0.03	Beta	(17)
Constipation	0.010	0.008	0.01	Beta	(17)
Arthralgia	0.013	0.010	0.02	Beta	(17)
Peripheral neuropathy	0.029	0.023	0.03	Beta	(17)
Vomiting	0.019	0.016	0.02	Beta	(17)
Hypertension	0.107	0.085	0.13	Beta	(17)
Urinary tract infection	0.081	0.065	0.10	Beta	(17)
Neutropenia	0.097	0.078	0.12	Beta	(17)
Peripheral sensory neuropathy	0.019	0.016	0.02	Beta	(17)
Thrombocytopenia	0.045	0.036	0.05	Beta	(17)
Terminal cost, US$					
Best supportive care	274.36	219.49	329.23	Gamma	Local data
End-of-life care	685.90	548.72	823.08	Gamma	Local data
Utility value					
Progression-free disease	0.76	0.61	0.91	Beta	(21)
Progressive disease	0.52	0.42	0.62	Beta	(22)
Disutility due to Grade ⩾3 AEs	-0.28	-0.22	-0.34	Beta	(23)
Discount rate	0.05	0	0.08	Beta	(24)

OS, overall survival; PFS, progression-free survival; ECG, electrocardiogram; CT, computed tomography; AE, adverse events; NA, Not Applicable.

For our study, Merck Sharp & Dohme Corp. was the manufacturer of pembrolizumab, Haikou Pharmaceutical Factory Co., Ltd. produced paclitaxel, Jiangsu Hossen Pharmaceutical Group Co., Ltd. produced cisplatin, and Shanghai Henlius Biotech Inc. produced bevacizumab. Drug pricing information was derived from the 2023 Drug Price Directory from the National Health Commission, with pembrolizumab priced at US$ 2,458/100 mg, paclitaxel at US$ 22/30 mg, cisplatin at US$ 3/30 mg, and bevacizumab at US$ 159/100 mg. The dosage and strength considerations were based on the findings of the KEYNOTE-826 trial. Because the trial did not specify body surface area or weight, we estimated the body surface area to be 1.62 m² and weight to be 59 kg using average demographic data for Chinese women from the “China Statistical Yearbook 2022,” published by the National Bureau of Statistics of China.

For our assessment, we adhered to a uniform schedule for both the laboratory and imaging examinations. During each treatment cycle, the patients were subjected to a series of tests: electrocardiogram (ECG), hematology, serum chemistry, urinalysis, and coagulation tests. Thyroid evaluations were conducted once every two cycles. For tumor imaging, the initial scan was scheduled for week 9, followed by assessments every 9 weeks up to week 54 and every 12 weeks thereafter. The imaging methodology, which included computed tomography (CT) of the chest and magnetic resonance imaging (MRI) of the abdomen and pelvis, was consistently applied in the study.

Our model emulated real-world drug administration logistics, accounting for costs, such as hospitalization, nursing care, and drug infusion. Drug wastage was factored in by rounding off drug quantities to the nearest vial size, aligning with the common practice of discarding surplus drugs after infusion. According to the KEYNOTE-826 trial, 63.6% of patients received bevacizumab in the pembrolizumab plus chemotherapy group, and 62.5% in the chemotherapy-only group. We used these proportions to estimate chemotherapy costs.

Treatment-associated AEs (grades 3–5) were factored into costs. We assumed that the AEs manifested during the initial treatment cycle (25, 26). Costs related to these AEs were determined by expert consensus. We also projected the costs for medical consultations on disease progression and end-of-life care by referencing the 2023 charging standards from the Fujian Provincial Health Commission.

Our projection incorporated costs associated with medical consultations regarding disease progression and end-of-life care. These terminal care expenses were obtained from the 2023 charging standards set by the Fujian Provincial Health Commission.

### 2.5 Utility scores

Utility scores measure the quality of life associated with specific health states. While the KEYNOTE-826 trial did not provide direct utility score data, scholars have previously employed quality of life data extracted from other studies as reference points for utility scores in cost-effectiveness analyses pertaining to cervical cancer treatments (27, 28). In our model, we derived these scores based on the recognized evaluations of cervical cancer utilities. Notably, the utility score was 0.76 for PFS, 0.52 for PD, and 0 for death (21, 22). Additionally, we factored in the adverse impact on the quality of life resulting from grade 3 or higher AEs, by representing these impacts as negative utility scores (23). Essential utility parameters are presented in **Table 1**.

### 2.6 Sensitivity analyses

We conducted a deterministic sensitivity analysis of our model by adjusting each input parameter across a range of ±20% to evaluate its individual impacts (29, 30). This involved altering a single parameter at a time while maintaining all others at their baseline values, thereby assessing the robustness of the model to specific parameteric changes. The annual discount rate was set at 5% for both costs and health outcomes, with a sensitivity range of 0–8% (24). Moreover, probabilistic sensitivity analysis was performed using Monte Carlo simulations (31, 32). Within this framework, cost parameters were assumed to adhere to a gamma distribution, and utility parameters followed a Beta distribution (33). In each of the 10,000 simulation iterations, all parameter values were randomly drawn from their designated distributions and applied simultaneously. The cumulative influence of these parameter fluctuations was then analyzed to determine the overall robustness of the model.

### 2.7 Subgroup analyses

For the subgroup analysis, we computed the ICER using subgroup-specific hazard ratios (HR) derived from the KEYNOTE-826 trial data. Owing to data limitations, we adopted methodologies from the existing literature, hypothesizing that the HRs for PFS within these subgroups paralleled those of the broader cohort (34). Subgroups were defined based on criteria such as age, race, ECOG performance status score, PD-L1 CPS, concurrent bevacizumab use, and the presence of metastatic disease at diagnosis. Given the sparse data, we employed proportional hazard assumptions for our evaluation. The analysis was predicated on a scenario in which the willingness-to-pay (WTP) threshold equaled three times China’s average Gross Domestic Product (GDP), translating to US$ 35,269.

### 2.8 Statistical analysis

We utilized the Get Data Graph Digitizer software (version 2.26 http://getdata-graph-digitizer.software.informer.com/) to extract survival curves from the KEYNOTE-826 trial data. Individual patient data were regenerated using the R software platform (version 4.2.2) to model patient survival rates across a range of distributions, namely the Weibull, log-normal, log-logistic, Gompertz, gamma, and exponential distributions. The best-fitting distribution was selected based on the lowest values for both the Akaike information criterion (AIC) and Bayesian information criterion (BIC). The choice was further influenced by visual assessments and insights from the existing literature. Following this rigorous evaluation, the log-Logistic distribution was selected to model the 5-year PFS for patients in both the pembrolizumab plus chemotherapy and chemotherapy-only groups. Similarly, the log-Logistic distribution was used to represent the 5-year OS for these groups. Computations related to costs, health outcomes across the three defined health states, and the subgroup and sensitivity analyses were conducted using Excel (version 2019).

## 3 Results

### 3.1 Base case analysis

Over the 5-year study period, the pembrolizumab combination group incurred a cost of US$ 159,113.44, while the standard chemotherapy group incurred US$ 24,610.88. This difference resulted in an incremental cost of US$ 134,502.57. The addition of pembrolizumab to the standard chemotherapy treatment resulted in a QALY increase of 1.18 (2.72 vs. 1.55) compared to that of the standard chemotherapy regimen alone. Consequently, the ICER for the pembrolizumab-enhanced regimen versus that for the standard chemotherapy regimen was estimated to be US$ 114,275.67/QALY. Given that pembrolizumab has not been integrated into China’s National Medical Insurance Directory, patients are inclined to access the drug through the charitable donation policy offered by Merck Sharp & Dohme Corp. This enabled them to acquire pembrolizumab at approximately 41.4% of its original price per dose. Under these conditions, the cost for the pembrolizumab combination group was adjusted to US$ 86,716.14, with the cost for the standard chemotherapy group remaining at US$ 24,610.88. This adjustment led to a revised incremental cost of US$ 62,105.26, representing a decrease from the initial incremental cost. However, the QALY increase remained consistent at 1.18 (2.72 vs. 1.55). Consequently, the recalculated ICER for the pembrolizumab-enhanced regimen in comparison to that of the standard chemotherapy regimen was US$ 52,765.69 per QALY. Further details are provided in **Table 2**.

**Table 2:** Results of the base-case analysis.

Treatment regimen	Total costs, US$	QALYs	ICER, US$/QALY
Original price-based analysis			
Pembrolizumab plus chemotherapy	159,113.44	2.72	114,275.67
Chemotherapy only	24,610.88	1.55	NA
Charitable donation policy price-based analysis			
Pembrolizumab plus chemotherapy	86,716.14	2.72	52,765.69
Chemotherapy only	24,610.88	1.55	NA

QALY, quality-adjusted life year; ICER, incremental cost-effectiveness ratio; NA, Not Applicable.

### 3.2 Sensitivity analyses

#### 3.2.1 Deterministic sensitivity analyses

**Figure 2:**
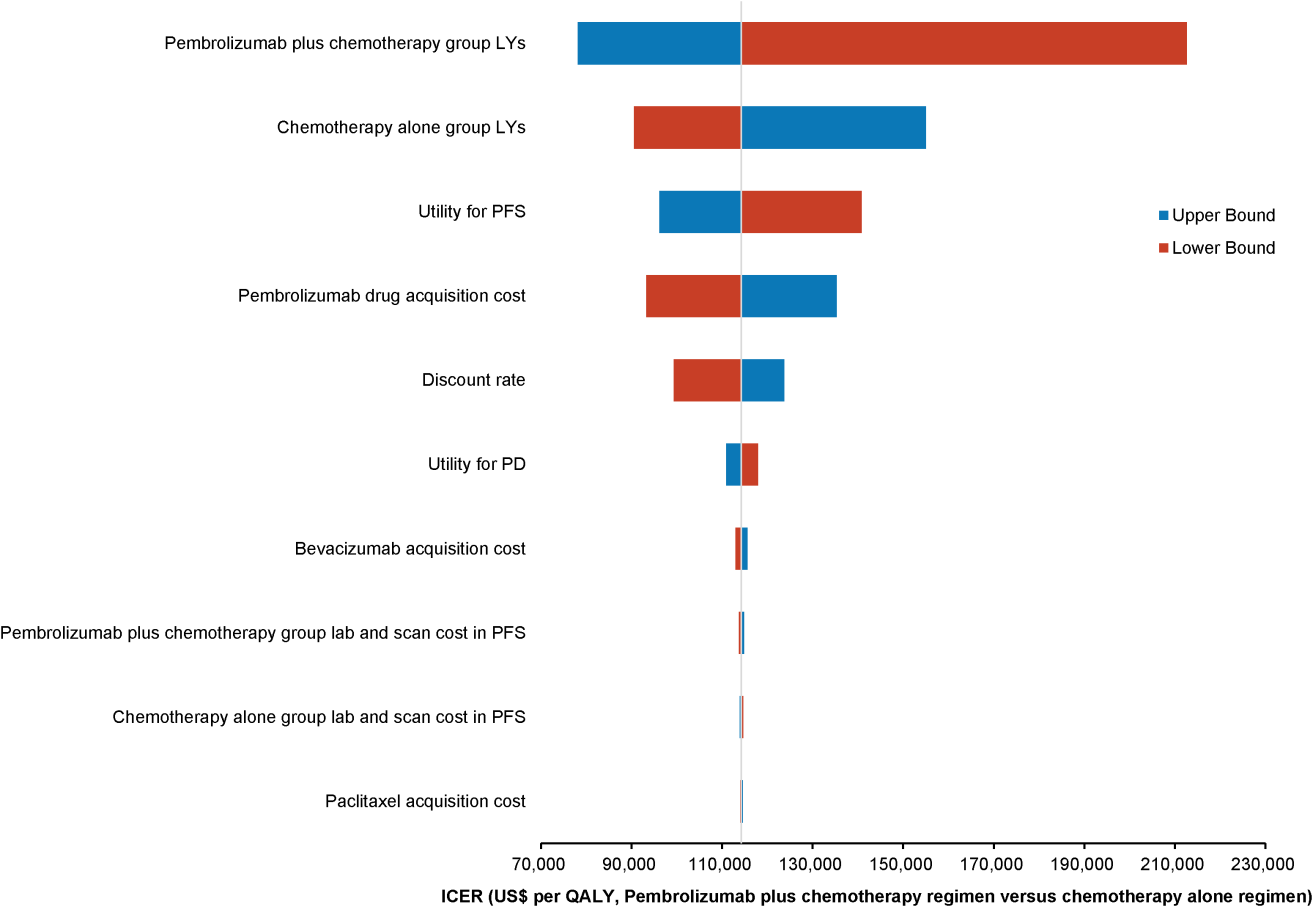
Tornado diagram illustrating the top 10 most influential parameters for pembrolizumab at the original pricing.

**Figure 3:**
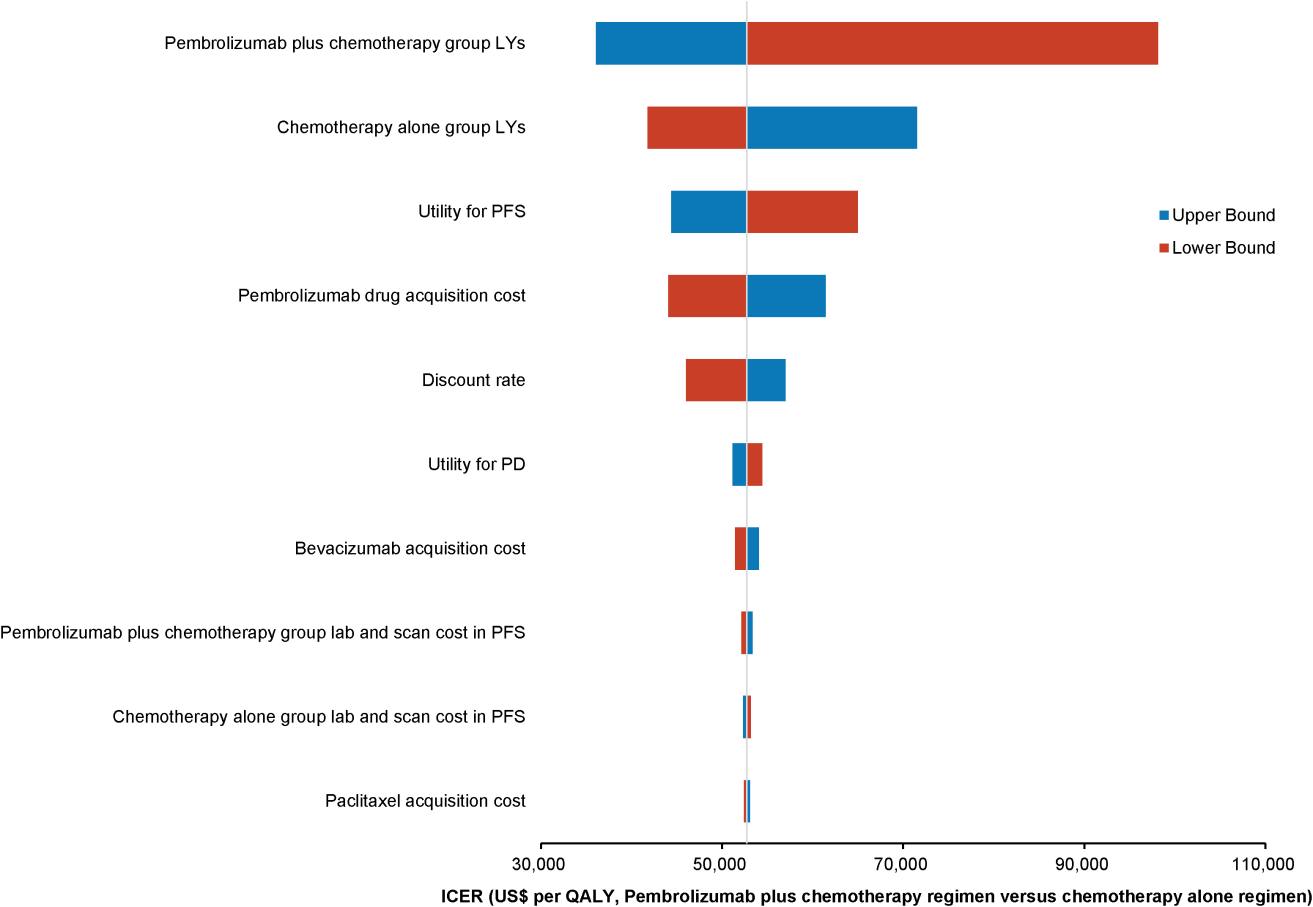
Tornado diagram showing the top 10 most influential parameters for pembrolizumab with Merck Sharp & Dohme Corporation’s charitable donation pricing.

One-way deterministic sensitivity analyses highlighted that the model was most sensitive to changes in the survival duration exhibited by the patients of the pembrolizumab combination group. Moreover, the survival duration of those in the standard chemotherapy group, utility for PFS, and cost of acquiring pembrolizumab were also significant factors influencing the model outcomes. The ten parameters exerting the most substantial influence on the outcomes are illustrated using a tornado diagram (**Figure 2**). Fluctuations in individual parameters led to a variations in ICER values, ranging from US$ 78,000 to US$ 213,000. Even when patients could obtain pembrolizumab at 41.4% of the original price through the donation policy offered by Merck Sharp & Dohme Corp, one-way deterministic sensitivity analyses revealed the model’s highest sensitivity concerning the survival duration of the pembrolizumab combination group. The ten most influential parameters remained consistent with those of the previous model and are depicted in the tornado diagram (**Figure 3**). However, in this adjusted model, variations in individual parameters caused the ICER values to range between US$ 36,100 and US$ 98,200.

#### 3.2.2 Probabilistic sensitivity analyses

**Figure 4:**
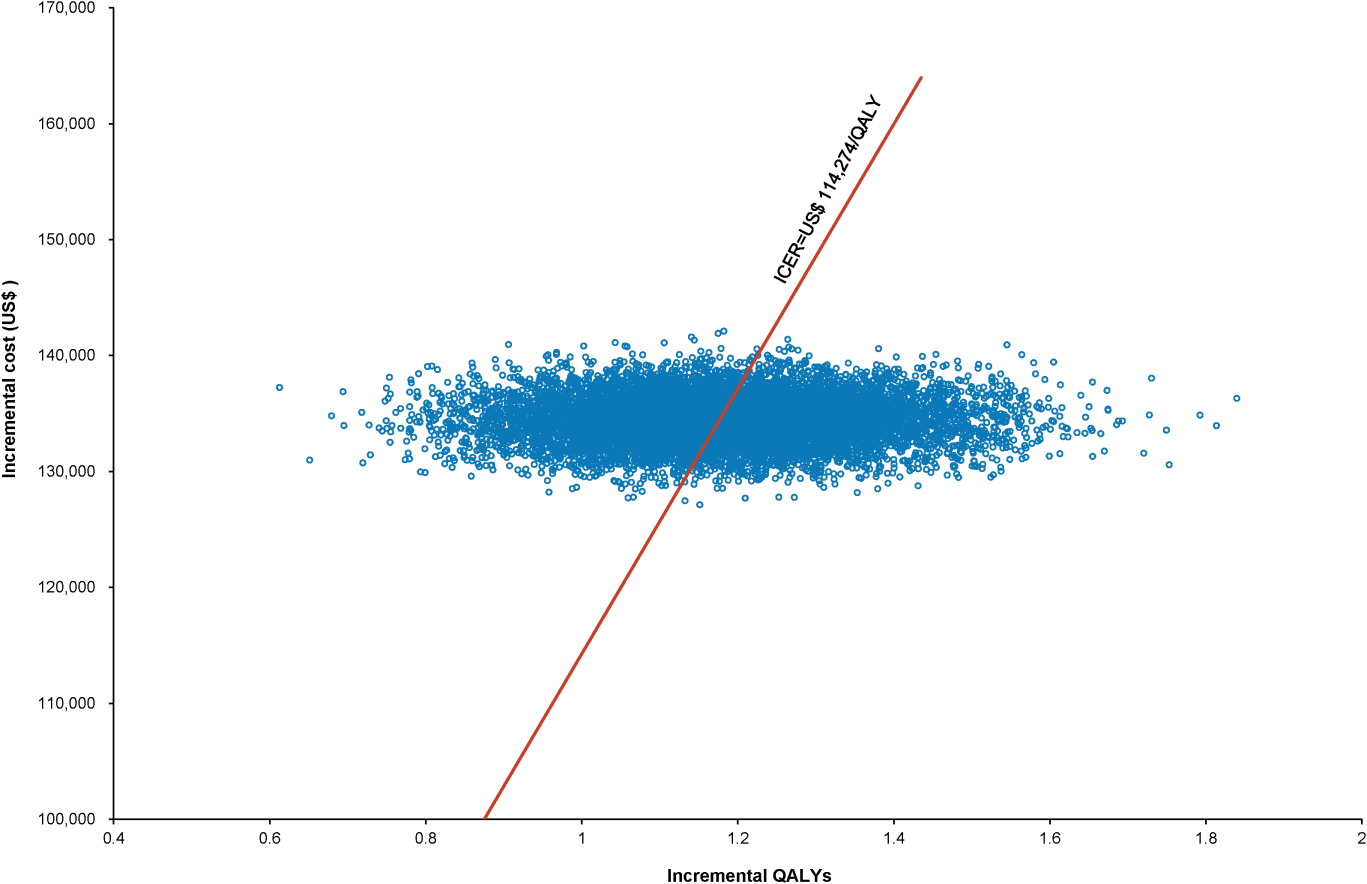
Scatter plot of Monte Carlo sensitivity analysis results for pembrolizumab at the original pricing.

**Figure 5:**
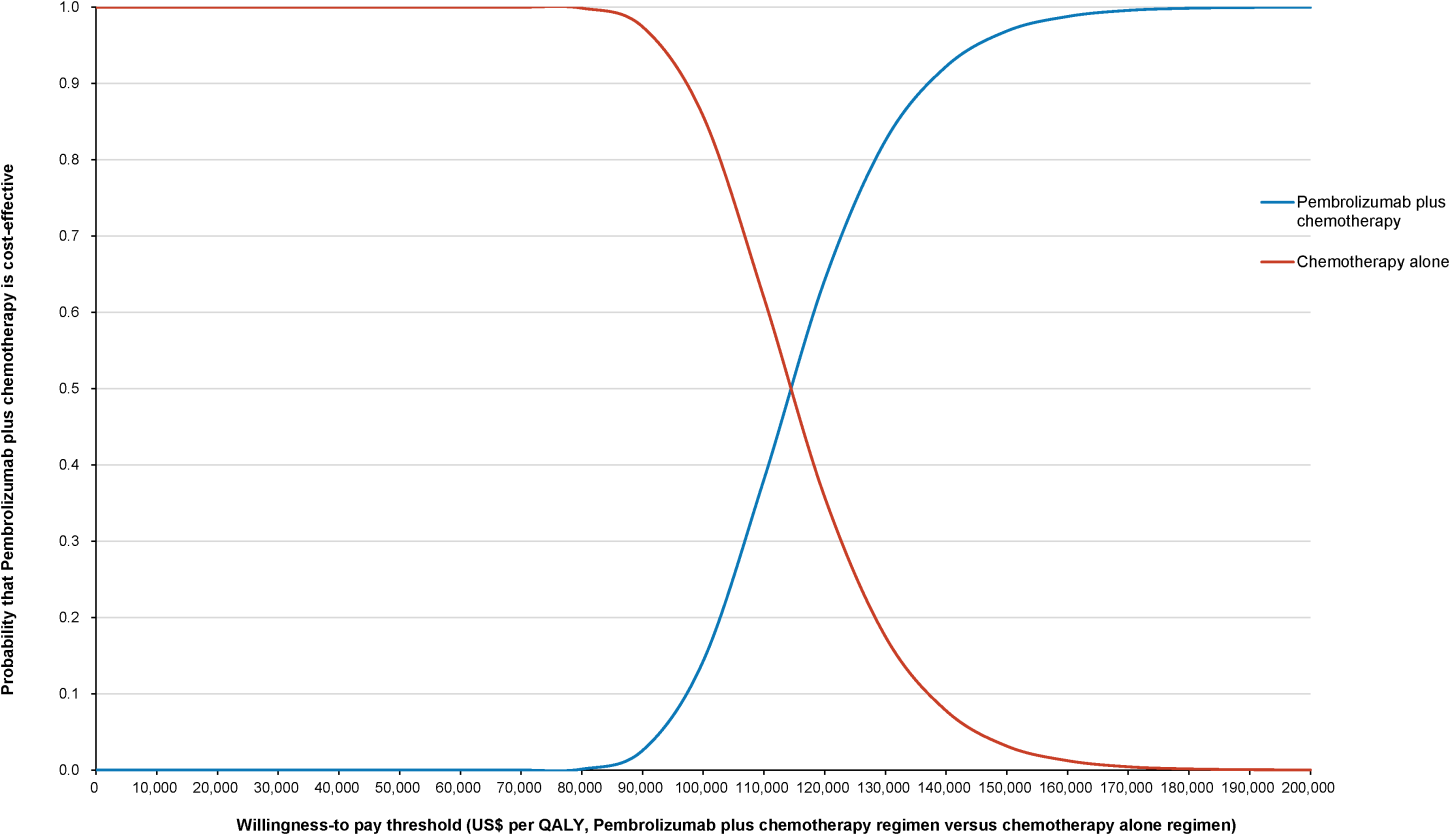
Cost-effectiveness acceptability curve for pembrolizumab plus standard therapy vs. standard therapy alone at the original pembrolizumab pricing.

**Figure 6:**
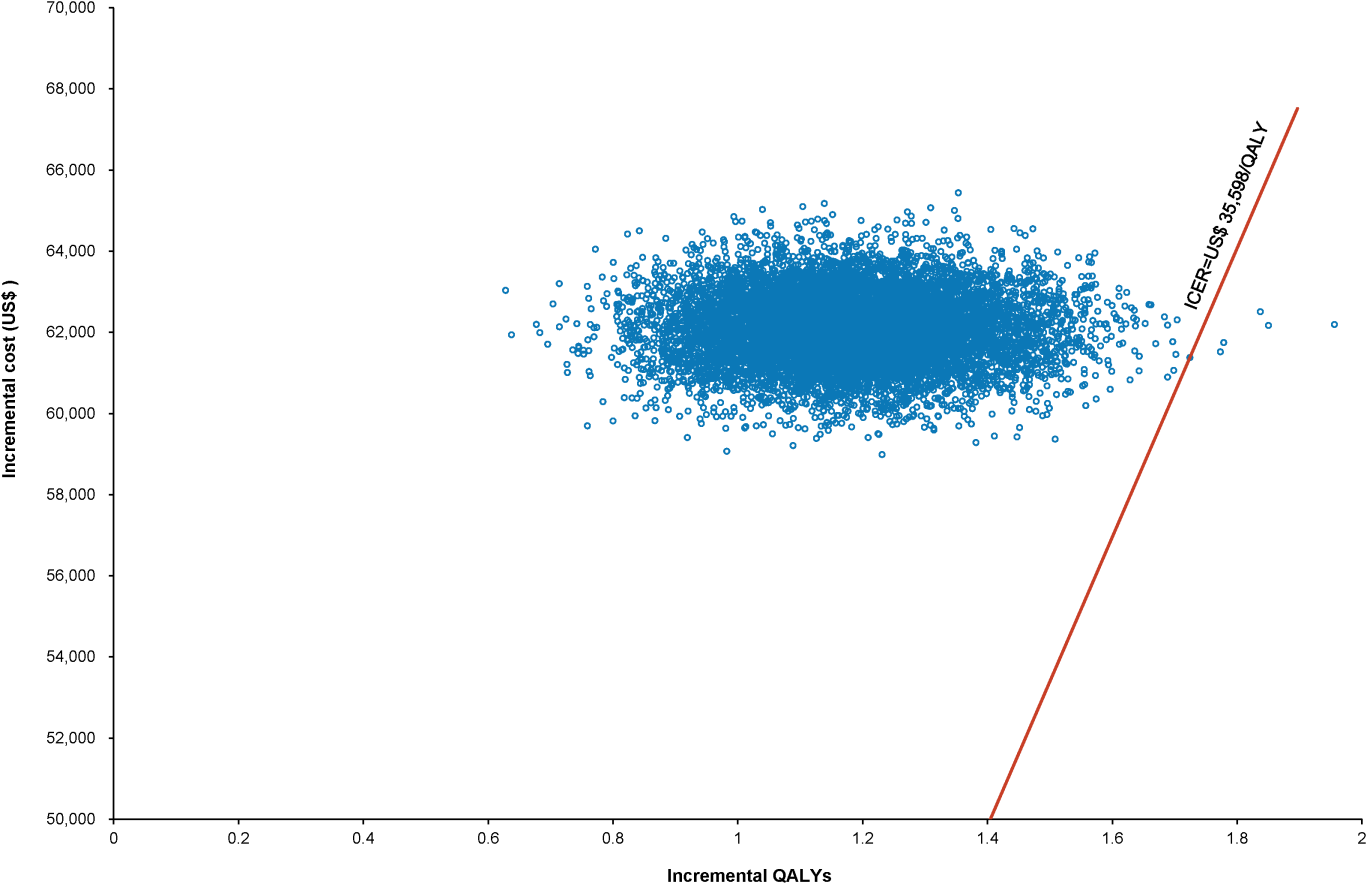
Scatter plot of Monte Carlo sensitivity analysis results for pembrolizumab under Merck Sharp & Dohme Corporation’s charitable donation pricing.

**Figure 7:**
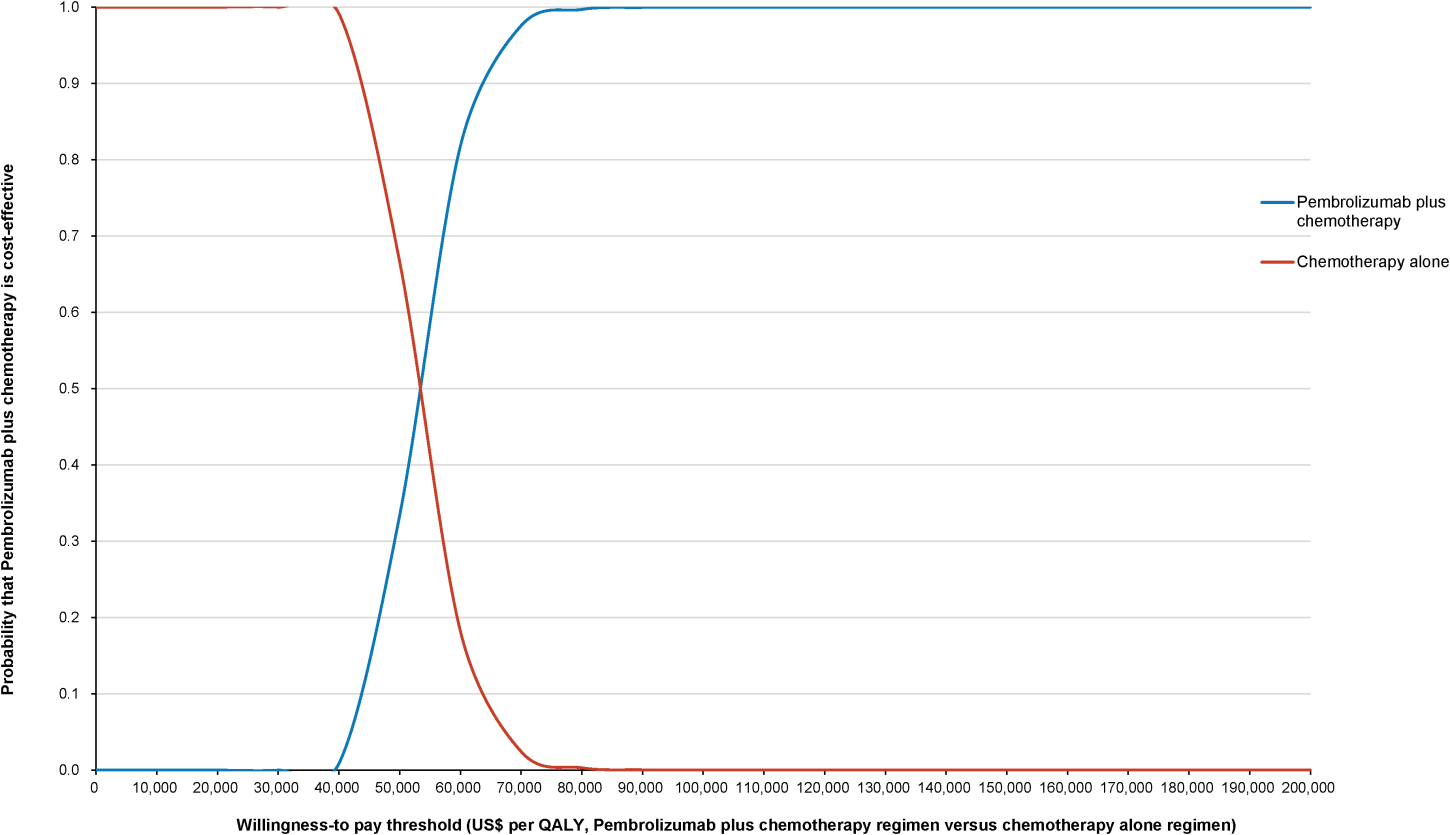
Cost-effectiveness acceptability curve for pembrolizumab plus standard therapy vs. standard therapy alone under Merck Sharp & Dohme Corporation’s charitable donation pricing.

The World Health Organization (WHO) advocates a WTP threshold set at three times the GDP per capita (35). As of 2022, China’s GDP per capita was US$ 11,756.31, which translated to a WTP threshold of US$ 35,268.94/QALY. Monte Carlo probabilistic sensitivity analyses indicated that at a WTP threshold of US$ 35,268.94/QALY, the pembrolizumab-combination therapy was not a cost-effective alternative to the standard chemotherapy regimen (as depicted in **Figures 4**, **5**). However, when factoring in the donation policy pricing for pembrolizumab, the probability of the pembrolizumab-combination therapy being cost-effective, in comparison to cost-effectiveness of the standard chemotherapy, increased to 0.6%. The simulated result closest to the WTP threshold was US $35,598, marginally above the US $35,268.94/QALY benchmark (**Figures 6**, **7**).

### 3.3 Subgroup analysis

In the subgroup analysis using a WTP threshold set at three times China’s GDP per capita, pembrolizumab combination therapy was not deemed cost-effective compared with the standard chemotherapy across all subgroups. However, when the donation policy pricing for pembrolizumab was considered, enhanced cost-effectiveness was observed in several subgroups: patients under 65 years (3.22%), white race individuals (0.10%), those with ECOG scores of 0 (0.17%) or 1 (0.11%), PD-L1 CPS of 1 to less than 10 (0.21%) and 10 or above (16.20%), patients receiving concomitant bevacizumab (6.20%), and those without metastatic disease at diagnosis (15.78%). **Table 3** presents the results.

**Table 3:** Subgroup analysis results for pembrolizumab access through Merck Sharp & Dohme Corporation’s charitable donation policy.

Subgroup	HR for OS (95%CI)	ICER, US$/QALY (range)	Cost-effectiveness probability of pembrolizumab plus chemotherapy, %
Age			
<65 years	0.64 (0.50-0.82)	43,751.67 (28,086.70, 88,954.48)	3.22%
≥65 years	0.88 (0.47-1.64)	121,407.90 (25,706.76, dominated)	0.00%
Race			
White	0.68 (0.50-0.91)	50,302.54 (28,086.70, 145,687.29)	0.10%
Non-White	0.70 (0.47-1.04)	54,098.86 (25,706.76, 56,8324.58)	0.00%
ECOG performance-status score			
0	0.68 (0.49-0.96)	50,302.54 (27,264.55, 210,595.47)	0.17%
1	0.68 (0.50-0.94)	50,302.54 (28,086.70, 179,532.24)	0.11%
PD-L1 combined positive score			
<1	1.00 (0.53-1.89)	312,773.04 30,744.50, dominated)	0.00%
1 to <10	0.67 (0.46-0.97)	48,543.80 (24,968.42, 229,916.59)	0.21%
≥10	0.61 (0.44-0.84)	39,580.89 (23,566.66, 98,045.05)	16.20%
Concomitant bevacizumab			
Yes	0.63 (0.47-0.87)	42,298.31 (25,706.76, 114,774.62)	6.20%
No	0.74 (0.53-1.04)	63,027.11 (30,744.50, 568,324.58)	0.00%
Metastatic disease at diagnosis			
Yes	0.84 (0.56-1.26)	98,045.05 (33,726.69, dominated)	0.00%
No	0.61 (0.46-0.80)	39,580.89 (24,968.42, 81,120.54)	15.78%

HR, hazard ratio; OS, overall survival; CI, confidence interval; ICER, incremental cost-effectiveness ratio; QALY, quality-adjusted life year; dominated, to indicate an absolute disadvantaged regimen; ECOG, Eastern Cooperative Oncology Group; PD-L1, programmed death-ligand 1.

## 4 Discussion

Our research suggests that, for patients with persistent, recurrent, or metastatic cervical cancer undergoing first-line therapy, the combination of pembrolizumab with chemotherapy or bevacizumab offers an additional 1.18 QALYs, compared with those estimated after chemotherapy alone or chemotherapy in conjunction with bevacizumab, over a 5-year period. However, this therapeutic approach increased the total cost by US$ 134,502.57, resulting in an ICER of US$ 114,275.67/QALY when juxtaposed with that from conventional chemotherapy regimens. A significant determinant of this outcome was the high price of pembrolizumab. Even after considering the 41.4% charitable discount extended by Merck & Co., the incremental cost of pembrolizumab diminished only to US$ 62,105.26, with the QALY increment maintained at 1.18. Consequently, the recalibrated ICER for the pembrolizumab-enhanced regimen stood at US$ 52,765.69/QALY relative to that of standard chemotherapy. In both the outlined scenarios, the ICER substantially surpassed China’s prevailing WTP threshold, which was set at three times the nation’s GDP per capita or US$ 35,268.94/QALY. Therefore, we posit that the financial burden of integrating pembrolizumab into the first-line treatment paradigm for persistent, recurrent, or metastatic cervical cancer might render it noncost-effective within the Chinese context. This stance is corroborated by studies of Barrington and Shi, who discerned that the pembrolizumab combination regimen may lack cost-effectiveness in the U.S. as well when set against traditional chemotherapy or bevacizumab protocols, by using their cost-effectiveness analysis models (36, 37).

In the management of cervical cancer, radical surgery is the primary treatment for early-stage patients, yet the choice of optimal surgical technique remains a subject of ongoing debate (11, 12). Recent high-quality evidence increasingly supports an open surgical approach as the preferred treatment for this cancer (13, 14). In advanced stages, cisplatin-based chemotherapy forms the cornerstone of systemic therapy and has demonstrated considerable efficacy. Nevertheless, the outlook for patients with advanced, recurrent, or metastatic cervical cancer, especially those exhibiting resistance to platinum-based chemotherapy, continues to be grim. The scarcity of effective second or later line treatments is a significant concern. Reflecting this, recent NCCN guidelines have incorporated various new drugs for managing recurrent or metastatic cervical cancer. Tisotumab vedotin, a significant breakthrough, has obtained accelerated Food and Drug Administration (FDA) approval for this indication (38). For human epidermal growth factor receptor 2 (HER2)-positive recurrent or metastatic cervical cancer, trastuzumab deruxtecan is now the treatment of choice, while larotrectinib is advised for patients with neurotrophic tyrosine receptor kinase (NTRK) fusion gene-positive tumors (39, 40). Among the array of novel therapeutics, immune checkpoint inhibitors, particularly pembrolizumab, have emerged as frontrunners due to their extensive recognition and robust evidence base.

PD-1 is an immune checkpoint located on T cells that plays a crucial role in modulating T-cell activation, thus ensuring self-tolerance (41). This mechanism allows cells expressing PD-L1 and PD-L2 to circumvent immune surveillance, facilitating immune evasion of tumors (42). Within the tumor microenvironment, PD-1 expression is predominantly observed in tumor-infiltrating immune cells, whereas PD-L1 is found on both antigen-presenting cells and tumor cells. The interaction between these proteins serves as a regulatory checkpoint, hindering the development of robust adaptive antitumor immune responses (43). The rationale behind anti-PD-1/PD-L1 agents is to counteract this tumor-imposed T-cell suppression, thereby potentiating T-cell-mediated tumor eradication and conferring therapeutic benefits (44). This innovation has catapulted pembrolizumab and other PD-1/PD-L1 inhibitors to the vanguard of cancer therapeutics over the last decade, challenging traditional treatment paradigms. The efficacy of these therapeutics transcends a wide array of malignancies, with some patients achieving prolonged survival and clinical remission. Nonetheless, the associated prohibitive costs may limit its broad use. Consequently, pharmaceutical entities must reconsider their pricing strategies to ensure enhanced market penetration. Notably, while the retail price for pembrolizumab in mainland China is US$ 2,458/100 mg, it is US$ 4,800/100 mg in the U.S. and US$ 3,594/100 mg in Hong Kong. Thus, in mainland China, the price of pembrolizumab is only 54.2% of that in the U.S. and 68.3% of that in Hong Kong, positioning it as a relatively cost-effective alternative on a global scale. However, the absence of pembrolizumab coverage under the national medical insurance scheme in China significantly limits its accessibility and adoption (45).

China has significantly expanded its reimbursement coverage of innovative drugs. Since 2017, more than 40 new oncological agents have been incorporated into the national medical insurance drug list (46). Many of these inclusions were facilitated through price negotiations, granting pharmaceutical companies with innovative pipelines accelerating market access opportunities. In the 2020 update of the medical insurance directory, there was a significant emphasis on promoting innovative oncology drugs, especially those targeting the PD-1/PD-L1 axis (47). The government anticipates pharmaceutical enterprises to substantially reduce the market prices of their premium drugs in exchange for inclusion in the national medical insurance scheme. In response, many domestic pharmaceutical entities have recalibrated their pricing strategies, optimizing for broader market access, thereby ensuring that a larger patient demographic benefits from these medications (48, 49). This recalibration, coupled with insurance negotiations, aims to achieve long-term value maximization (50, 51).

Notably, the domestic PD-1 immune checkpoint inhibitors camrelizumab, toripalimab, and sintilimab have undergone significant price reductions through these insurance negotiations, with prices set at US$ 353/200 mg, US$ 147/100 mg, and US$ 148/100 mg, respectively. Although these PD-1 inhibitors have not yet received approval for cervical cancer indications, numerous clinical studies have demonstrated the therapeutic potential of these cost-effective agents in advanced cervical cancer. For instance, the CLAP study led by Dr. Huang and Dr. Lan of the Gynecology Department at Sun Yat-sen University Cancer Center evaluated the efficacy and safety of camrelizumab combined with apatinib in second-line or later treatments for advanced cervical cancer, reporting an objective response rate (ORR) of 55.6% and a median PFS of 8.8 months (52). Similarly, a clinical trial by our team, led by Dr. Xu, combined sintilimab with anlotinib for the treatment of recurrent metastatic cervical cancer, achieving an ORR of 54.9%, median PFS of 9.4 months, and 12-month OS rate of 73.8% (53). Moreover, the approval of domestic cadonilimab and zimberelimab for the treatment of recurrent or metastatic cervical cancer may pose a potential challenge to pembrolizumab in the near future (54).

Immune checkpoint inhibitors targeting PD-1 or PD-L1 have significant therapeutic potential for a variety of cancers. However, a mere 20–40% of patients derive tangible benefits from these treatments (55). Our subgroup analysis indicated that patients with a PD-L1 CPS of 10 or above and those not initially diagnosed with metastatic disease were notably more inclined to achieve cost-effective results than their counterparts in other subgroups. At present, PD-L1 expression levels are determined through immunohistochemical assays and serve as pivotal biomarkers for directing treatment involving anti-PD-1 or anti-PD-L1 antibodies.

The robust association between the therapeutic efficacy of PD-1/PD-L1 inhibitors and PD-L1 expression intensities has been highlighted in previous research. For instance, a Phase I/II study on durvalumab revealed that patients with advanced urothelial carcinoma, expressing PD-L1 in tumor or immune cells at 25% or more, reported heightened response rates (56). Furthermore, a meta-analysis of five Phase III randomized controlled trials on esophageal cancer showed that once the PD-L1 CPS is 10 or above, there is a marked augmentation in the patients’ PFS (57). In parallel, the KEYNOTE-158 study, centered on cervical cancer treatment, revealed that patients with advanced cervical cancer with prevalent PD-L1 expression had an overall response rate of 12.2% after pembrolizumab treatment (58). This compelling evidence facilitated the expedited FDA endorsement of pembrolizumab for addressing patients with recurrent or metastatic cervical cancer with PD-L1 positivity. Insights from the KEYNOTE-826 study further fortified the intrinsic connection between PD-L1 expression scales and the effectiveness of pembrolizumab (17). Thus, we speculate that across-the-board cost reduction of pembrolizumab for all patients with recurrent or metastatic cervical cancer may not be imperative. Instead, a tailored price adjustment for patients exhibiting a PD-L1 CPS ≥10 could amplify both therapeutic results and economic efficiencies for this specific advanced cervical cancer demographic.

We focused on the cost-effectiveness of pembrolizumab combined with chemotherapy and bevacizumab for the treatment of persistent, recurrent, or metastatic cervical cancer within the Chinese healthcare system in this study. We concluded that while the combination of pembrolizumab, chemotherapy, and bevacizumab offers clinical benefits, its high cost renders it a non-cost-effective primary strategy for advanced cervical cancer in the Chinese context. However, this stance might change for patients with a PD-L1 CPS ≥10, where price adjustment could improve both therapeutic and economic outcomes. The study results also highlighted the potential of pembrolizumab as a therapeutic agent for cancer. A tailored price adjustment for patients with advanced cervical cancer and a high PD-L1 CPS could be beneficial. Nevertheless, larger, multicenter, real-world studies are needed to assess the cost-effectiveness of pembrolizumab.

This study has several limitations that warrant consideration. First, while our model is not an inaugural attempt to evaluate the economic outcomes of pembrolizumab treatment for advanced cervical cancer, we aim to refine existing research findings. Second, our analysis primarily hinges on data from clinical trials, which could introduce subtle discrepancies and uncertainties into the results. Notably, the long-term therapeutic efficacy of pembrolizumab in combination with chemotherapy and bevacizumab for advanced cervical cancer remains under investigation. An extended follow-up could be instrumental in updating relevant data, offering clearer insights into this domain. Additionally, our model did not fully account for certain AE-related costs, especially those related to grade 3–5 AEs with incidences below 20% and common grade 1–2 AEs. Although these omissions might induce minor variations in the outcomes, our sensitivity analysis suggested that these factors might not substantially alter the primary conclusions within the defined variability range. The significance of utility values in pharmacoeconomic research must be emphasized. We relied on published utility values associated with metastatic cervical cancer, in the absence of direct QOL data from pertinent trials. While our univariate sensitivity analysis considered the potential influence of PFS and PD utility values on the outcomes, the insights from the tornado diagram suggest that the ICER is unlikely to fall below the WTP threshold, even with adjustments within acceptable bounds.

To conclude, the combination of pembrolizumab with chemotherapy and bevacizumab may not currently represent an economically viable first-line treatment alternative to the standard chemotherapy plus bevacizumab regimen for recurrent or metastatic cervical cancer. However, for patients with advanced cervical cancer and a CPS ≥10, targeted price adjustment for pembrolizumab could potentially improve both therapeutic outcomes and cost-effectiveness.

## Data availability statement

The original contributions presented in the study are included in the article/supplementary material. Further inquiries can be directed to the corresponding author.

## Author contributions

Y-TL: Conceptualization, Formal analysis, Methodology, Software, Writing – original draft, Writing – review & editing. CW: Data curation, Software, Writing – review & editing. X-YH: Data curation, Visualization, Writing – review & editing. Q-MY: Formal analysis, Software, Writing – review & editing. JC: Investigation, Supervision, Validation, Writing – review & editing.
